# Dietary Cameroonian Plants Exhibit Anti-Inflammatory Activity in Human Gastric Epithelial Cells

**DOI:** 10.3390/nu12123787

**Published:** 2020-12-10

**Authors:** Achille Parfait Atchan Nwakiban, Marco Fumagalli, Stefano Piazza, Andrea Magnavacca, Giulia Martinelli, Giangiacomo Beretta, Paolo Magni, Armelle Deutou Tchamgoue, Gabriel Agbor Agbor, Jules-Roger Kuiaté, Mario Dell’Agli, Enrico Sangiovanni

**Affiliations:** 1 Department of Biochemistry, Faculty of science, University of Dschang, P.O. Box 96 Dschang, Cameroon; achilestyle@yahoo.fr or jrkuiate@yahoo.com (J.-R.K.); 2 Department of Pharmacological and Biomolecular Sciences, Università degli Studi di Milano, 20133 Milan, Italy; marco.fumagalli3@unimi.it (M.F.); stefano.piazza@unimi.it (S.P.); andrea.magnavacca@unimi.it (A.M.); giulia.martinelli@unimi.it (G.M.); paolo.magni@unimi.it (P.M.); enrico.sangiovanni@unimi.it (E.S.); 3 Department of Environmental Science and Policy, Università degli Studi di Milano, 20133 Milan, Italy; giangiacomo.beretta@unimi.it; 4 IRCCS MultiMedica, Sesto San Giovanni, Via Milanese, 300, Sesto San Giovanni, 20099 Milan, Italy; 5 Centre for Research on Medicinal Plants and Traditional Medicine, Institute of Medical Research and Medicinal Plants Studies, 4124 Yaoundé, Cameroon; armelle_d2002@yahoo.fr (A.D.T.); agogae@yahoo.fr (G.A.A.)

**Keywords:** gastric inflammation, ethnopharmacology, Cameroonian plants, interleukin 8, interleukin 6, NF-κB, COX-2, antioxidant

## Abstract

In Cameroon, local plants are traditionally used as remedies for a variety of ailments. In this regard, several papers report health benefits of Cameroonian spices, which include antioxidant and anti-microbial properties, whereas gastric anti-inflammatory activities have never been previously considered. The present study investigates the antioxidant and anti-inflammatory activities of hydro-alcoholic extracts of eleven Cameroonian spices in gastric epithelial cells (AGS and GES-1 cells). The extracts showed antioxidant properties in a cell-free system and reduced H_2_O_2_-induced ROS generation in gastric epithelial cells. After preliminary screening on TNFα-induced NF-κB driven transcription, six extracts from *Xylopia parviflora*, *Xylopia aethiopica*, *Tetrapleura tetraptera*, *Dichrostachys glomerata*, *Aframomum melegueta*, and *Aframomum citratum* were selected for further studies focusing on the anti-inflammatory activity. The extracts reduced the expression of some NF-κB-dependent pro-inflammatory mediators strictly involved in the gastric inflammatory process, such as IL-8, IL-6, and enzymes such as PTGS2 (COX-2), without affecting PTGS1 (COX-1). In conclusion, the selected extracts decreased pro-inflammatory markers by inhibiting the NF-κB signaling in gastric cells, justifying, in part, the traditional use of these spices. Other molecular mechanisms cannot be excluded, and further studies are needed to better clarify their biological activities at the gastric level.

## 1. Introduction

Gastritis is an inflammatory-based pathology which can be classified as acute or chronic. Acute gastritis is provoked by several risk factors which include stress, alcohol abuse, the use of non-steroidal anti-inflammatory drugs (NSAIDs), and bile reflux; chronic conditions are mainly due to *Helicobacter pylori* (*H. pylori*) infection. Infected people can also develop more severe pathologies, such as peptic ulcer or gastric cancer [1,2]. In 1994, the WHO assessed *H. pylori* as a class I carcinogen for gastric cancer. Several transcription factors are involved in gastric inflammatory conditions, including NF-κB [3]; according to the literature, in vitro studies demonstrate that *H. pylori* and some pro-inflammatory cytokines (i.e., TNFα) are able to induce the activity of this transcription factor in gastric epithelial cells [4,5]. NF-κB plays a pivotal role in the expression and release of some pro-inflammatory mediators, such as IL-8, IL-6 and enzymes such as prostaglandin endoperoxide synthase 2 (PTGS2) (COX-2), which, in turn, lead to the amplification of the gastric phlogistic process [6,7]. IL-8 is considered a key element involved in the development of gastritis. At this regard, Crabtree et al. demonstrated the increased expression of this chemokine in the epithelium of the infected gastric mucosa [8,9]. In this context, an over-production of oxygen reactive species (ROS) leads to increased oxidative stress contributing to exacerbate the inflammatory process [10]. Indeed, it has been demonstrated that several ROS can enhance IL-8 expression in gastric epithelial cells through the NF-κB activation [11].

NSAIDs act on prostaglandin synthesis, through the inhibition of cyclooxygenase enzymes, PTGS1 (COX-1) and PTGS2 (COX-2). The latter is encoded by an NF-κB-dependent gene, strictly involved in the inflammatory process, whose expression can be rapidly up-regulated by cytokines and growth factors; otherwise, *PTGS1* gene is constitutively expressed in human epithelial cells, contributing to preserve the protective mucus layer by cytoprotective prostaglandin production; therefore, the blockage of PTGS1 (COX-1) activity is the main factor responsible for the gastric side effects (e.g., peptic ulcers) of NSAID chronic drug treatment [12,13,14]. These findings underline the importance of selective inhibitor agents for COX-2, able to preserve COX-1 activity.

In Cameroon, the traditional use of natural products for the treatment of several diseases is largely diffused and constitutes the first health approach among the population [15]. The efficacy of the traditional use of plants for human health is not generally fully supported by scientific evidence. Many plants are traditionally employed in Cameroon for the treatment of different ailments, such as diabetes, hypertension, malaria, and gastric disorders [16,17,18]. Nkui and Nahpoh are two traditional soups of the eastern region which contain many spices, among which there are plants used in the present study. These plants are widely distributed in eastern and central Africa, and their fruits and seeds, normally boiled with the help of a thread or a stick of bamboo, are pounded and used in cooked foods or as spices for sauces or beverages. As a traditional remedy, they are mainly employed in association with other botanicals for the treatment of various ailments, including stomach disorders [18,19,20]. According to the literature, several papers report the potential health benefits of these Cameroonian spices, including anti-microbial, anti-inflammatory, and hypoglycemic properties [19,21,22,23]. However, no study has investigated the anti-inflammatory activity of Cameroonian spices at the gastric level.

The aim of this study is to investigate the potential anti-inflammatory and antioxidant effects of the hydroethanolic extracts from eleven selected Cameroonian spices in human gastric epithelial cells. Human GES-1 and AGS cells are used as reliable in vitro models of human gastric epithelium. The extracts are assessed on the TNFα-induced expression of different NF-κB-dependent mediators, such as IL-8, IL-6 and PTGS2 (COX-2), and the effect on the TNFα-induced NF-κB-driven transcription is assessed as well. Among the extracts tested, six showed promising activity as anti-inflammatory agents, confirming their use in Cameroonian traditional medicine for the treatment of gastric disorders.

## 2. Materials and Methods

### 2.1. Preparation of the Hydroethanolic Extracts

The material from eleven different species of plants was harvested in different areas of the West Cameroon in September 2017, as previously described [24,25]. Plants collected were: *Xylopia aethiopica* (Dunal) A.Rich. (**XA**), *Xylopia parviflora* Spruce (**XP**), *Scorodophloeus zenkeri* Harms (**SZ**), *Monodora myristica* (Gaertn.) Dunal (**MM**), *Tetrapleura tetraptera* (Schum. and Thonn.) Taub. (**TT**), *Echinops giganteus* A. Rich. (**EG**), *Dichrostachys glomerata* (Forssk.) Chiov. syn. *Dichrostachys cinerea* (L.) Wight and Arn. (**DG**), *Afrostyrax lepidophyllus* Mildbr. (**AL**), *Aframomum melegueta* K.Schum. (**AM**), *Aframomum citratum* (C. Pereira) K. Schum. (**AC**), and *Zanthoxylum leprieurii* Guill. and Perr. (**ZL**).

Selected samples consisted of different fruits, seeds, or roots, and were identified in the National Herbarium of Cameroon (http://irad.cm/national-herbarium-of-cameroun/) in Yaoundè (Cameroon), by comparison with preserved specimens [24,25]. The plant material (100 g) of each species was powdered and extracted, under stirring, with 100 mL of an hydroalcoholic (ethanol:water, 70:30) mixture for 4 h at room temperature, in dark conditions. Then, the extract was filtered, and the plant material was recovered and subjected to a second overnight extraction with fresh solvent. The solvent was removed through rotary evaporator (Laborota 4000 efficient, Heidolph Instruments, Schwabach, Germany), and subjected to lyophilization. All the extracts were dissolved in pure DMSO, aliquoted, and stored at −80 °C. Parts used, aspect, color, and extraction yield were previously reported in [25]. The extracts were characterized by gas chromatography coupled with mass spectrometry, as previously reported [24].

### 2.2. ORAC Assay

The oxygen radical absorbance capacity (ORAC) assay was carried out according to Ou B. et al. and Dávalos A. et al. [26,27], with minor modifications. Briefly, 20 μL of stock solution of each extract (1 μg/mL) was distributed into a black 96-well plate. Then, 120 µL of fluorescein solution (70 nM final concentration), previously prepared with a phosphate buffer (pH 7.4, 75 mM), was added to each well. Peroxyl radicals were generated by adding 60 µL of AAPH 40 mM (Sigma-Aldrich, St. Louis, MO, USA). The final concentration of each extract in the assay well was 0.1 μg/mL. The plate was put in a spectrophotometer (Victor X3, PerkinElmer, USA) and the fluorescence detector was set at excitation and emission wavelengths of 484 and 528 nm, respectively. The fluorescence was read, after shaking, every 2 min for 60 min at 37 °C. Trolox (0–50 μM) was used as reference inhibitor. The area under the curve (AUC), of each extract, was calculated and the results were expressed as μM Trolox equivalent.

### 2.3. Cell Culture

Human adenocarcinoma gastric epithelial cells (AGS, CRL-1739, LGC Standard S.r.l., Milano, Italy) were grown in DMEM/F12 medium (Gibco, Thermo Fisher Scientific, Waltham, MA, USA) supplemented with penicillin 100 units/mL (Gibco, Thermo Fisher Scientific, Waltham, MA, USA), streptomycin 100 mg/mL (Gibco, Thermo Fisher Scientific, Waltham, MA, USA), L-glutamine 2 mM (Gibco, Thermo Fisher Scientific, Waltham, MA, USA) and 10% heat-inactivated fetal bovine serum (Euroclone S.p.A, Pero, Italy), at 37 °C in humidified atmosphere containing 5% CO_2_. Human normal gastric epithelial cells (GES-1, kindly provided by Dr. Dawit Kidane-Mulat (University of Texas, Austin) were grown in RPMI 1640 medium, supplemented as previously described, and in the same atmospheric conditions. When cells reached 80–90% of confluence, usually every 4 days, they were detached from the flask (Euroclone S.p.A., Milan, Italy) using trypsin-EDTA 0.25% (Gibco, Thermo Fisher Scientific, Waltham, MA, USA), counted, and replaced in a new flask (1 × 10^6^ cell density per flask) to promote cell growth.

### 2.4. Cytotoxicity Assay

Cell morphology was checked by light microscope inspection. Cell viability was measured, after 6 h co-treatment with the stimulus (TNFα, 10 ng/mL) and the extracts, by the 3,4,5-dimethylthiazol-2-yl-2,5-diphenyltetrazolium bromide (MTT) method (Sigma-Aldrich, St. Louis, MA, USA) [28]. This method evaluates the succinate dehydrogenase activity, which is an index of cell viability. Six hours later, the medium was removed from each well and 0.1 mg/mL of MTT solution (200 μL) was added for 45 min at 37 °C, in dark conditions. Violet formazan salt was extracted from the cells with 200 μL of a mixture isopropanol:DMSO (90:10), and the absorbance was read at 570 nm (Envision, PerkinElmer, Waltham, MA, USA). The extracts were assessed in the range 0.1–20 μg/mL.

### 2.5. Cell Treatment

To investigate the release and gene expression of the pro-inflammatory mediators, the NF-κB driven transcription, and IL-8 promoter activity, cells were seeded in 24-well plates (DB Falcon^TM^) at the density of 30,000 cells/well. After 72 h, cells were co-treated with the pro-inflammatory stimulus (TNFα, 10 ng/mL) and the extracts for 6 h, using serum-free medium: DMEM/F12 or RPMI 1640 medium (Gibco, Thermo Fisher Scientific, Waltham, USA), supplemented with L-glutamine 2 mM (Gibco, Thermo Fisher Scientific, Waltham, USA), penicillin 100 units/mL (Gibco, Thermo Fisher Scientific, Waltham, MA, USA), and streptomycin 100 mg/mL (Gibco, Thermo Fisher Scientific, Waltham, USA). Then, the medium or the cell lysate was collected for the biological assays. To assess the effect of the extracts on ROS generation, AGS and GES-1 cells were seeded in black 96-well plates (PerkinElmer, USA) at the density of 10,000 cells/well; after 72 h, cells were pre-treated with the extracts for 24 h in serum-free medium, and subsequently challenged with H_2_O_2_ for 2 h.

### 2.6. ROS Production

The intracellular ROS level was measured, in GES-1 and AGS cells, with the oxidant-sensitive fluorescence probe CM-H2DCFDA (Invitrogen, Thermo Fisher Scientific, Waltham, MA, USA). After the treatment, cells were incubated for 30 min with the fluorescent probe (10 μM), washed with PBS and stimulated with H_2_O_2_ for 2 h. The fluorescence intensity was read at an excitation wavelength of 485 nm and an emission wavelength of 535 nm using a plate reader (Envision, Perkin Elmer, Waltham, MA, USA). Trolox (500 μM) was used as reference compound. Data were expressed as mean ± SD of at least three experiments.

### 2.7. Transient Transfection Assays

GES-1 and AGS cells were transiently transfected in 24-well plates with two different reporter plasmids, the NF-κB-Luc (50 ng/well) or the IL-8-Luc (100 ng/well) [29]. The NF-κB-Luc contains the luciferase gene under control of the E-selectin promoter characterized by three κB responsive elements, while IL-8-Luc contains the luciferase gene under control of a fragment of the native promoter of the human IL-8. The plasmid NF-κB-Luc was a kind gift of Dr. N. Marx (Department of Internal Medicine-Cardiology, University of Ulm, Ulm, Germany)m whereas the native IL-8-Luc promoter was kindly provided by Dr. T. Shimohata (Department of Preventive Environment and Nutrition, University of Tokushima Graduate School, Tokushima, Japan). GES-1 cells were transfected using Lipofectamine^®^ (Invitrogen, Thermo Fisher Scientific, Waltham, MA, USA), whereas AGS cells by the calcium phosphate method. Sixteen hours later, the cells were treated for 6 h with extracts in the presence of the pro-inflammatory mediators (TNFα 10 ng/mL). Six hours later, cells were harvested and the luciferase assay was carried out using the Britelite^TM^ Plus reagent (PerkinElmer Inc., Waltham, MA, USA), according to the manufacturer’s instructions. The results (mean ± SD of at least three experiments) were expressed as percentage, relative to stimulated control, which was arbitrarily assigned the value 100%.

### 2.8. IL-8 and IL-6 Release

IL-8 and IL-6 were quantified in cell media after the treatment with TNFα and plant extracts, using two different ELISA kits, a Human Interleukin-8 ELISA Development Kit and a Human Interleukin-6 ELISA Development Kit (Peprotech Inc., London, UK). Briefly, Corning 96-well EIA/RIA plates (Sigma-Aldrich, St. Louis, USA) were coated with the corresponding antibody provided by the ELISA Kit (Peprotech Inc., London, UK) and incubated overnight at room temperature. The non-specific binding sites were blocked with albumin 1%. A total of 200 μL of samples was transferred in duplicate into wells at room temperature for 2 h. The amount of IL-8 and IL-6 in the samples was detected at 450 nm by measuring the colorimetric reaction between horseradish peroxidase enzyme and 3,3′,5,5′-tetramethylbenzidine substrate (Sigma-Aldrich, St. Louis, MO, USA). The absorbance was read at 450 nm using a spectrophotometer (Victor X3, PerkinElmer, USA). IL-8 and IL-6 were quantified by optimization of the standard curve provided with the ELISA kit (8.0–1000 pg/mL for IL-8 and 32–2000 pg/mL for IL-6). Epigallocatechin-3-O-gallate (EGCG, 20 μM) was used as a reference molecule able to inhibit TNFα-induced IL-8 and IL-6 secretion. The results (mean ± SD of at least three experiments) were expressed as percentage, relative to stimulated control, which was arbitrarily assigned the value 100% (values around 1000 and 1500 pg/mL, respectively for IL-8 and IL-6 under stimulated conditions).

### 2.9. Gene Expression

GES-1 and AGS cells were lysed with QIAzol Lysis Reagent (Qiagen, Hilden, Germany), RNA was purified using the miRNeasy Mini Kit (Qiagen, Hilden, Germany) and quantified by spectrophotometric analysis at 260 nm (NanoDrop ND-100, Thermo Fisher Scientific, Waltham, MA, USA). Primers for *IL-8*, *IL-6*, *PTGS1* (*COX-1*), *PTGS2* (*COX-2*) and *GAPDH* (housekeeping gene) genes Are reported in Table S1. *IL-8, IL-6, PTGS1* (*COX-1*), and *PTGS2* (*COX-2*) mRNA levels were investigated through SYBR Green method (iTaq Universal SYBR Green One-Step Kit, Bio-Rad Laboratories Srl, Segrate, Italy). The total RNA (10 ng/μL) from each sample was mixed with SYBR Green, corresponding primers and the reverse transcription enzyme, the real-time PCR was assessed using the CFX384^TM^ Real-Time PCR Detection System (coupled to C1000^TM^ Thermal Cycler; Bio-Rad Laboratories Srl, Segrate, Italy). The threshold values were set manually, the relative expression of each gene was calculated by normalizing the data on the basis of the housekeeping gene (GAPDH). The experiments were repeated, at least, three times.

### 2.10. Statistical Analysis

All results are expressed as mean (±SD) of at least three experiments. Data were analyzed by unpaired one-way analysis of variance (ANOVA), followed by Bonferroni post hoc test. Gene expression results were calculated using the ΔΔCt method. ΔΔCt values were analyzed by unpaired t-test or unpaired one-way analysis of variance (ANOVA), followed by Bonferroni post hoc test, and graphed as mean relative expression (2^ΔΔCt) values (± SEM). Statistical analyses were calculated using GraphPad Prism 8.0 software (GraphPad Software Inc., San Diego, CA, USA) as well as IC_50_s. *p* < 0.05 was considered statistically significant.

## 3. Results

### 3.1. Cytotoxicity of the Extracts in Gastric Epithelial Cells

The cytotoxicity of the extracts was assessed in the concentration range 0.1–20 μg/mL in both human gastric epithelial cells (GES-1 and AGS cells) by means of the MTT assay. After 6 h treatment, XA extract showed cytotoxic effects only at 20 μg/mL in both the cell cultures (data not shown), and this concentration was not used for assessing the biological activity of the extract.

### 3.2. Hydroalcoholic Extracts Inhibit ROS Production in AGS and GES-1 Cells

**Figure 1:**
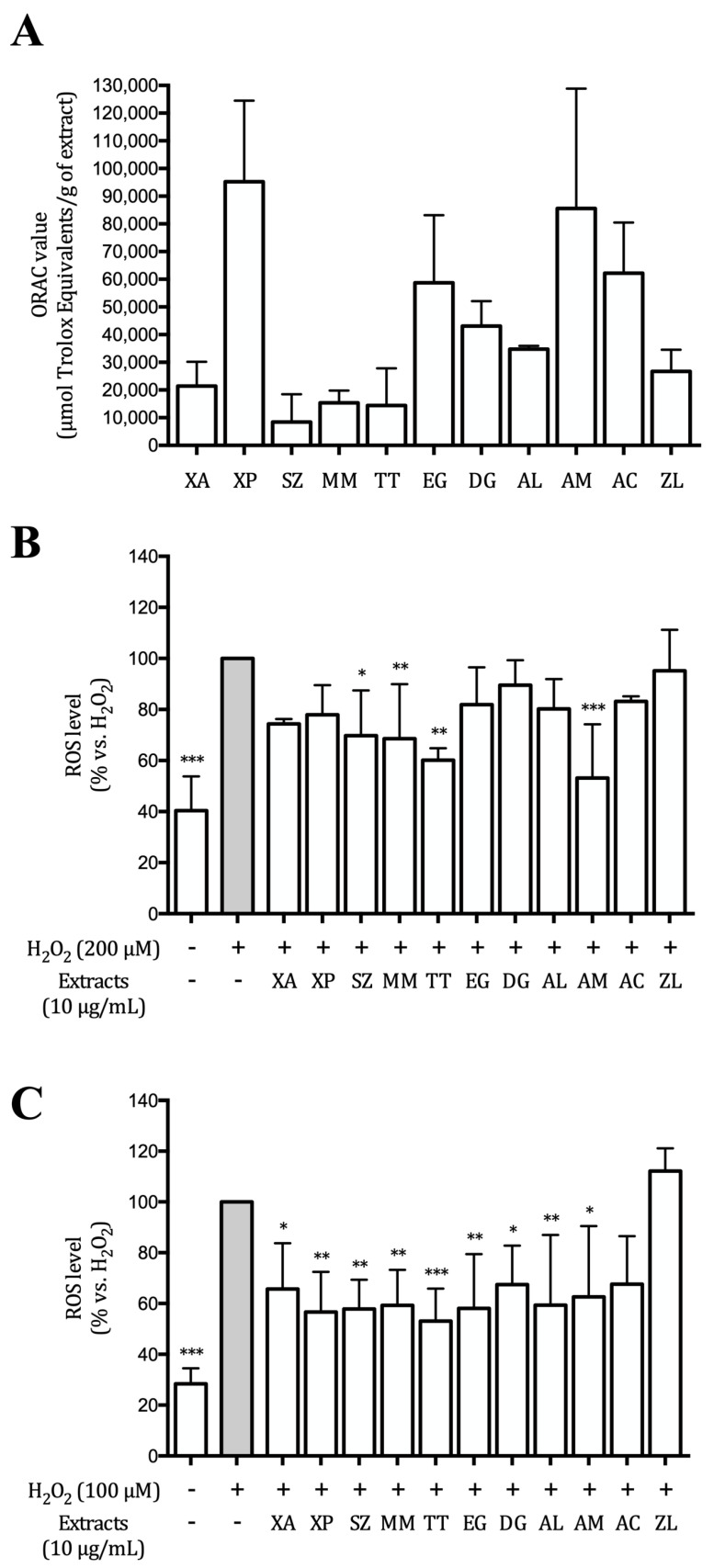
Antioxidant activity of the extracts. Oxygen radical absorbance capacity (ORAC) assay (**A**). Data are expressed as ORAC value (µmol Trolox Equivalent/g of sample). Effect of the extracts on intracellular ROS production induced by hydrogen peroxide in human gastric adenocarcinoma (AGS) (**B**) and gastric epithelial (GES-1) (**C**) cells. Antioxidant activity is expressed as μmol Trolox equivalent. Data reported in panels B and C are expressed as percentage versus the stimulated control (grey bar), to which is arbitrarily assigned the value 100%. * *p* < 0.05; ** *p* < 0.01; *** *p* < 0.001.

The extracts were investigated for their ability to block peroxyl radicals in a cell-free system (ORAC) assay, and to reduce ROS generation in AGS and GES-1 cells. XP and AM extracts were the most active in ORAC assay (around 9.5 and 8.5 μmol Trolox equivalents, respectively), followed by EG and AC extracts (around 6 μmol Trolox equivalents) (Figure 1A).

ROS generation in AGS and GES-1 cells stimulated with hydrogen peroxide (200 μM in AGS and 100 μM in GES-1 cells) was close to 2.5-fold and 3.5-fold compared to the unstimulated controls, respectively. Most of the extracts inhibited ROS production induced by H_2_O_2_.

SZ, MM, TT, and AM exhibited a statistically significant inhibition in AGS cells (*p* < 0.05, Figure 1B), while in GES-1 cells the effects were more pronounced since all the extracts, except for ZL, showed a significant effect approximately halving the levels of ROS (Figure 1C).

### 3.3. Effect of the Extracts on the TNFα-Induced NF-κB-Driven Transcription in AGS and GES-1 Cells

**Figure 2:**
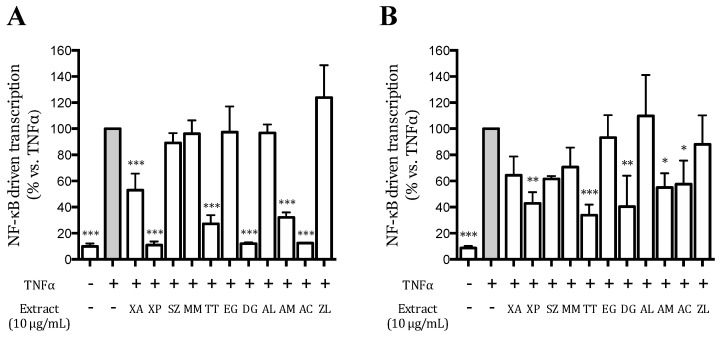
Effect of the extracts (10 μg/mL) on the NF-κB-driven transcription in human gastric adenocarcinoma (AGS) (**A**) and gastric epithelial (GES-1) (**B**) cells. Data are expressed as percentage versus the stimulated control, which is arbitrarily assigned the value 100% (grey bar). * *p* < 0.05; ** *p* < 0.01; *** *p* < 0.001.

The eleven hydroalcoholic extracts were preliminary screened at 10 μg/mL for their ability to impair the TNFα-induced NF-κB driven transcription, in AGS and GES-1 cells. TNFα increased the NF-κB driven transcription around 10-folds compared to unstimulated cells, in both cell lines (Figure 2); XP, TT, DG, AM, and AC extracts significantly impaired the activation in both cell lines, while XA only in AGS cells. The effect was more pronounced in AGS than in GES-1 cells.

XA, XP, TT, DG, AM, and AC extracts were further investigated for their ability to inhibit the TNFα-induced NF-κB driven transcription in a concentration-dependent manner (Figures S1 and S2). The extracts were tested in the range 0–10 μg/mL, and the corresponding IC_50_ values were calculated. In AGS cells, all the extracts, except for XA, showed an IC_50_ below 10 μg/mL (the highest concentration tested) and DG was the most active (IC_50_ 2.1 μg/mL). In GES-1 cells, the IC_50_s were higher compared to those obtained in AGS cells, and only TT and DG extracts showed IC_50_ values below 10 μg/mL (Table 1).

### 3.4. Plant Extracts Inhibit TNFα-Induced IL-8 Release and Expression in AGS and GES-1 Cells

IL-8 is a well-known NF-κB-dependent chemokine strictly involved in the inflammatory process at the gastric level [5,7]; for this reason, the selected extracts (XP, XA, TT, DG, AM, and AC) were assessed for their ability to reduce its release as well as its promoter activity induced by TNFα in AGS and GES-1 cells. All the extracts, at concentrations ranging between 0.1 and 10 μg/mL, were able to reduce IL-8 secretion with different IC_50_s (Figures S3 and S4), whose values were lower in AGS cells compared to GES-1 cells, as previously observed on the NF-κB driven transcription. The extracts also inhibited the IL-8 promoter activity, in AGS and GES-1 cells (Figures S5 and S6), suggesting that the effects showed on IL-8 release could be due, at least in part, to the impairment of the promoter activity (Table 2).

Since IC_50_s on IL-8 release were not always comparable with those on promoter activity, we investigated the inhibitory mechanism at mRNA level; concentrations corresponding to the IC_50_ values obtained on IL-8 release by each extract were tested for this purpose.

**Figure 3:**
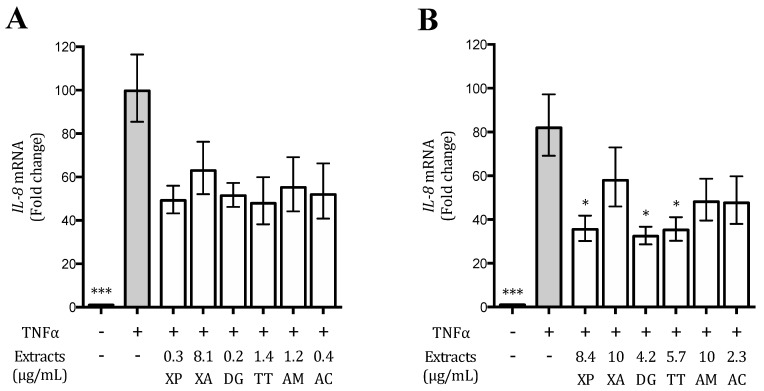
Effect of the extracts on *IL-8* mRNA levels in human gastric adenocarcinoma (AGS) (**A**) and gastric epithelial (GES-1) (**B**) cells. Data are expressed as fold changes versus stimulated control (grey bar). * *p* < 0.05; *** *p* < 0.001.

In GES-1 cells, the extracts with an IC_50_ >10 μg/mL (XA and AM) were tested at 10 μg/mL. As shown in Figure 3, in AGS cells, the *IL-8* mRNA levels were around 40–50% lower with respect to stimulated control; however, this effect was not statistically significant. In GES-1 cells, XP, DG, and TT extracts were able to reduce the *IL-8* mRNA levels of around 60%, while the activity of AC extract was around 40% lower. These results confirmed that the activity on IL-8 release could be attributed to an effect at transcriptional level (Figure 3).

### 3.5. Effect of the Extracts on the TNFα-Induced IL-6 Release and Expression in AGS and GES-1 Cells

IL-6 is a cytokine dependent on the NF-κB activation involved in *H. pylori*-induced gastric inflammation [30]. Thus, the possible ability of the extracts to counteract gastric inflammation through the decrease of IL-6 release and expression was investigated as well. In these studies, just GES-1 cells were considered since TNFα did not induce any detectable IL-6 secretion in AGS cells (data not shown). The extracts were tested on IL-6 release in the range 0–10 μg/mL (Figure S7), while the activity on *IL-6* gene expression was screened at 10 μg/mL. XP and DG extracts inhibited IL-6 release both with an IC_50_ of 3.5 μg/mL, whereas the effect of TT and AC was lower (4.9 and 5.1 μg/mL, respectively); XA and AM exhibited an IC_50_ higher than 10 μg/mL (Table 3).

**Figure 4:**
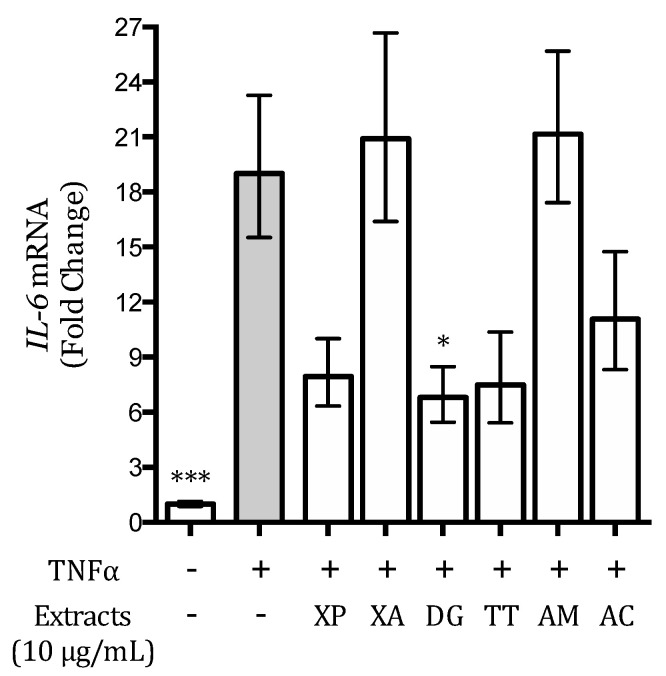
Effect of the extracts on IL-6 mRNA levels in gastric epithelial (GES-1) cells. Results are expressed as fold changes versus stimulated control (grey bar). * *p* < 0.05; *** *p* < 0.001.

The effect of DG extract (10 μg/mL) on *IL-6* gene expression was statistically significant, as displayed in Figure 4; XP, DG, and TT also reduced *IL-6* mRNA levels; however, this effect was not statistically significant. XA and AM extracts did not show any activity (Figure 4).

### 3.6. Tetrapleura Tetraptera Extract Inhibits PTGS2 (COX-2) Gene Expression in AGS and GES-1 Cells

**Figure 5:**
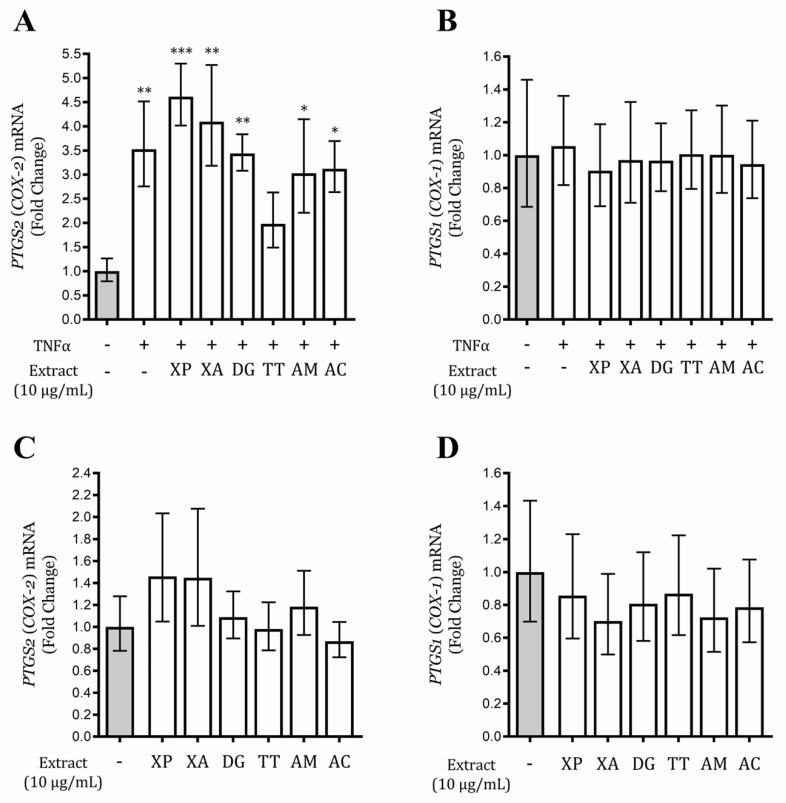
Effect of the extracts on prostaglandin endoperoxide synthase 2 *PTGS2* (*COX-2*) and prostaglandin endoperoxide synthase 1 *PTGS1* (*COX-1*) mRNA levels in the presence (**A**,**B**) or in the absence (**C**,**D**) of TNFα, in gastric epithelial (GES-1) cells. Data are expressed as fold changes versus control (grey bar). * *p* < 0.05; ** *p* < 0.01; *** *p* < 0.001.

The gene expression, induced by TNFα, of the two cyclooxygenases, *PTGS1* (*COX-1*) and *PTGS2* (*COX-2*), was investigated at different times (1, 3, and 6 h) in AGS and GES-1 cells. *PTGS1* (*COX-1*) expression, as expected, was not induced by the stimulus in both cell lines; *PTGS2* (*COX-2*) mRNA was considerably increased in GES-1 cells at 6 h, while a mild increased after 1 h stimulation was observed in AGS cells (data not shown). The activity of the extracts on the gene expression of the two isoforms of cyclooxygenase was evaluated in GES-1 cells after 6 h treatment, in basal and inflammatory conditions. The *PTGS2* (*COX-2*) mRNA level of the cells treated with TT extract and TNFα was the only condition non-statistically different with respect to unstimulated control (Figure 5), suggesting the ability of this extract to reduce TNFα-induced *PTGS2* (*COX-2*) gene expression; the effect was exerted without affecting the gene expression of the *PTGS1* (*COX-1*) isoform. TT extract was also inactive on *PTGS2 (COX-2*) and *PTGS1* (*COX-1*) mRNA levels in the basal conditions (Figure 5). Interestingly, TT extract did not affect the enzymatic activity of PTGS1 (COX-1) and PTGS2 (COX-2) isoforms in a cell-free system (data not shown).

## 4. Discussion

Gastritis is an inflammatory-based condition promoted by a variety of risk factors, which include stress, alcohol or drug abuse, and others; the most severe chronic form of gastritis is mainly caused by *Helicobacter pylori* infection. Unfortunately, the infection is diffused in several countries with unsustainable economic situation, and the conventional therapy against gastric inflammatory processes or for *H. pylori* eradication is not easily available.

This work investigates the anti-inflammatory and radical scavenger activities at the gastric level of eleven hydroalcoholic extracts obtained from plants widely used in Cameroon as spices and in the traditional medicine against a variety of diseases, including gastric disorders.

The study was carried out using two gastric cell models (AGS and GES-1 cells) stimulated with TNFα, which contributes to the inflammatory process in infected gastric epithelium [31]. AGS cells, a tumor gastric epithelial cell line, is a well-established in vitro model, while GES-1 cells are considered an in vitro model closer to the gastric epithelium of healthy subjects [32].

A prolonged oxidative stress may cause lipid peroxidation and DNA damages, leading to an increased risk to develop gastric cancer [33,34]; several extracts included in this study prevented H_2_O_2_-induced ROS generation in AGS and GES-1 cells, this effect could be explained with the presence of compounds able to counteract the excessive amount of ROS, derived from the unbalance between ROS production and endogenous antioxidant systems, acting as scavengers. The extracts displaying the highest antioxidant effect were those with the highest phenol content, as previously reported [25].

The investigation on the anti-inflammatory activity identified six extracts (XA, XP, DG, TT, AM, and AC) able to reduce the release and the gene expression of two NF-κB-dependent pro-inflammatory mediators, IL-8 and IL-6, which contribute to the amplification of the gastric inflammatory process [6,8,31]; the effect on the NF-κB pathway suggests that this transcription factor could be involved in the molecular mechanism responsible for the observed activity. In addition, the IC_50_s on IL-8 release in our experiments reflected in halved IL-8 mRNA level, suggesting the important contribution of the transcription, although not always statistically confirmed. The different activity on the IL-8 promoter, especially evident in GES-1 cells, may be partly justified by the intervention of post-transcriptional regulatory mechanisms. The chemical analysis of the extracts has been previously reported by our group [24]; on the basis of this characterization, the hydroalcoholic extracts contain previously quantified secondary metabolites able to act as NF-κB inhibitors, such as pimaric acid in XP (8.73%), gingerol and shogaol in AM (0.7% and 2.05% respectively), chlorogenic acid and catechins, identified in some extracts [35,36,37,38,39,40]. Although further investigation is needed, these individual compounds could contribute, at least in part, to the anti-inflammatory activity displayed by the extracts. According to the literature, the anti-inflammatory effects could be linked to the antioxidant properties of the extracts, since it has been demonstrated that ROS are able to over-express IL-8 by activating oxidant-sensitive nuclear factors, such as NF-κB, in gastric epithelial cells [11].

Although the six extracts inhibited IL-8 release in both gastric cell models, only XP, TT, DG, and AC reduced IL-6 levels. Again, the mRNA analysis showed that all the extracts influenced the IL-6 at the transcriptional level, in particular DG, which obtained a statistically significant difference with respect to the stimulated control. Our results allow to speculate that XA and AM do not inhibit other important transcription factors for IL-6, such as CREB, but further studies are needed to confirm this hypothesis.

NSAIDs are not used in the therapy of gastric inflammatory diseases, since they act on prostaglandin-endoperoxide synthase (cyclooxygenase) enzymes blocking also the PTGS1 (COX-1) isoform, which is involved in the protection of the gastric mucosa [14]. In this context, TT extract could be considered the most promising extract, for the ability to inhibit TNFα-induced *PTGS2* (*COX-2*) gene expression without affecting the *PTGS1* (*COX-1*) mRNA basal levels. Indeed, *Tetrapleura tetraptera* (TT) extract did not reduce the enzymatic activity of PTGS1 (COX-1) and PTGS2 (COX-2) isoforms in a cell-free system; however, these findings need further investigation.

In the literature, different studies have demonstrated the beneficial effects of the fruits of this plant including antimalarial, anti-inflammatory, hypotensive, anti-insulin resistance, and antilipidemic properties [22,41,42].

This study demonstrates the antioxidant and anti-inflammatory activity, at the gastric level, of different hydroalcoholic extracts from Cameroonian plants, selecting those with the most promising effects. XA, XP, TT, DG, AM, and AC are largely used and diffused in some African countries as spices, thus making these plants interesting as functional foods, especially XP, TT, DG, and AC. Further studies are clearly needed to collect more evidence, especially for TT extract, although our findings provide a sound scientific support to the traditional use of these plants against gastric disorders. In particular, studies on the stability of the extracts in the gastric environment should reinforce the translational value of this work. Considering the order of the inhibitory concentrations (0.1–10 μg/mL) and the yields of extraction [23], it is plausible that the dietary consumption of 1–10 mg of Cameroonian spices may achieve the bioactivity at gastric level. Thus, these products could be useful to alleviate gastric inflammation in countries where conventional therapy is not easily available.

## Figures and Tables

**Table 1:** Half-maximal Inhibitory Concentration (IC_50_) (μg/mL) of the extracts on the TNFα-induced NF-κB driven transcription in human gastric adenocarcinoma (AGS) and gastric epithelial (GES-1) cells.

	AGS Cells	GES-1 Cells
	IC_50_ (μg/mL)	
*Xylopia parviflora* Spruce (XP)	4.1	>10
*Xylopia aethiopica* (Dunal) A. Rich. (XA)	>10	>10
*Tetrapleura tetraptera* (Schum. and Thonn.) Taub (TT)	9.7	5.9
*Dichrostachys glomerata* (Forssk.) Chiov. (DG)	2.1	8.8
*Aframomum melegueta* K.Schum (AM)	9.9	>10
*Aframomum citratum* (C.Pereira) K.Schum (AC)	6.8	>10

**Table 2:** Half-maximal Inhibitory Concentration (IC_50_) (μg/mL) of the extracts on the TNFα-induced IL-8 release and expression in human gastric adenocarcinoma (AGS) and gastric epithelial (GES-1) cells.

	AGS Cells		GES-1 Cells	
	IC_50_ (μg/mL)			
	IL-8 release	IL-8 promoter activity	IL-8 release	IL-8 promoter activity
*Xylopia parviflora* Spruce (XP)	0.3	1.0	8.4	>10
*Xylopia aethiopica* (Dunal) A. Rich. (XA)	8.1	>10	>10	>10
*Tetrapleura tetraptera* (Schum. and Thonn.) Taub (TT)	1.4	1.4	5.7	4.6
*Dichrostachys glomerata* (Forssk.) Chiov. (DG)	0.2	0.3	4.2	>10
*Aframomum melegueta* K.Schum (AM)	1.2	1.9	>10	>10
*Aframomum citratum* (C.Pereira) K.Schum (AC)	0.4	2.0	2.3	>10

**Table 3:** Half-maximal Inhibitory Concentration (IC_50_) (μg/mL) of the extracts on the IL-6 release in gastric epithelial (GES-1) cells.

	GES-1 Cells IC_50_ (μg/mL)
*Xylopia parviflora* Spruce (XP)	3.5
*Xylopia aethiopica* (Dunal) A. Rich. (XA)	>10
*Tetrapleura tetraptera* (Schum. and Thonn.) Taub (TT)	4.9
*Dichrostachys glomerata* (Forssk.) Chiov. (DG)	3.5
*Aframomum melegueta* K.Schum (AM)	>10
*Aframomum citratum* (C.Pereira) K.Schum (AC)	5.1

## Supplementary Materials

The following are available online at https://www.mdpi.com/2072-6643/12/12/3787/s1, Figure S1: Effect of the extracts on the NF-κB driven transcription in AGS cells. Data are expressed as percentage versus the stimulated control, which is arbitrarily assigned the value 100%., Figure S2: Effect of the extracts on the NF-κB driven transcription in GES-1 cells. Data are expressed as percentage versus the stimulated control, which is arbitrarily assigned the value 100%, Figure S3: Effect of the extracts on the IL-8 release in AGS cells. Data are expressed as percentage versus the stimulated control, which is arbitrarily assigned the value 100%, Figure S4: Effect of the extracts on the IL-8 release in GES-1 cells. Data are expressed as percentage versus the stimulated control, which is arbitrarily assigned the value 100%, Figure S5: Effect of the extracts on the IL-8 promoter activity in AGS cells. Data are expressed as percentage versus the stimulated control, which is arbitrarily assigned the value 100%, Figure S6: Effect of the extracts on the IL-8 promoter activity in GES-1 cells. Data are expressed as percentage versus the stimulated control, which is arbitrarily assigned the value 100%, Figure S7: Effect of the extracts on the IL-6 release in GES-1 cells. Data are expressed as percentage versus the stimulated control, which is arbitrarily assigned the value 100%, Table S1: List of the primers used in the study.
