# Supplementary material for: Dietary Cameroonian Plants Exhibit Anti-Inflammatory Activity in Human Gastric Epithelial Cells
Source: Nutrients. 2020 Dec 10;12(12):3787. doi: 10.3390/nu12123787 (PMC7763248; doi:10.3390/nu12123787)
Supplement: Supplementary file 1 [file nutrients-12-03787-s001.pdf]

Table 1. List of the primers used in the study.

Gene	Forward primer	Reverse primer
IL-8	ATACTCCAAACCTTTCCACCC	TCTGCACCCAGTTTTCCTTG
IL-6	GGGAACGAAAGAGAAGCTC	AGGCAACTGGACCGAA
PTGS1 (*COX-1*)	TTCACCCACTTCCTGCT	GTGCTGAGTTGTAGGTGG
PTGS2 (*COX-2*)	GCTGGAACATGGAATTACCC	TCTGGTCAATGGAAGCCT
GAPDH	CGGGGCTCTCCAGAACATC	ATGACCTTGCCCACAGCCT

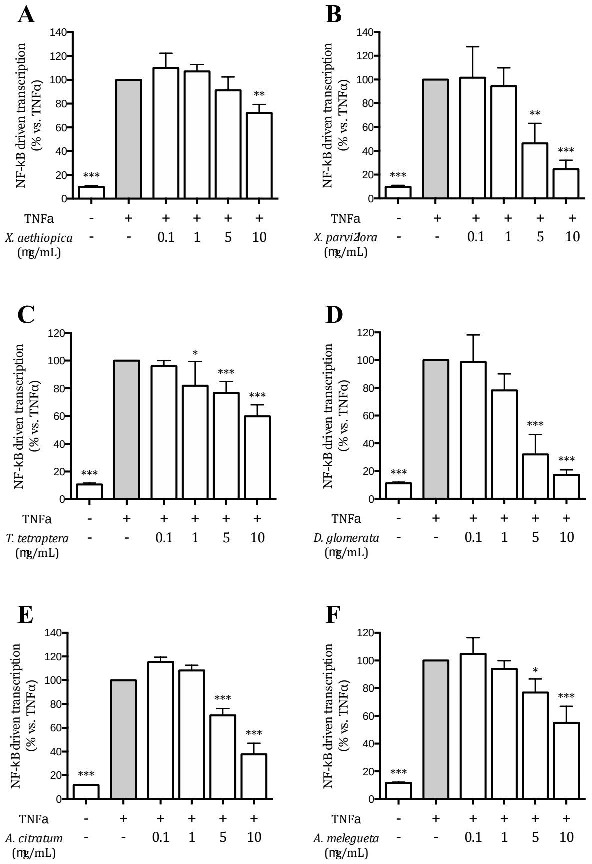

Figure 1. Effect of the extracts on the NF-κB driven transcription in AGS cells. Data are expressed as percentage versus the stimulated control, which is arbitrarily assigned the value 100%. * $p < 0.05$; ** $p < 0.01$; *** $p < 0.001$.

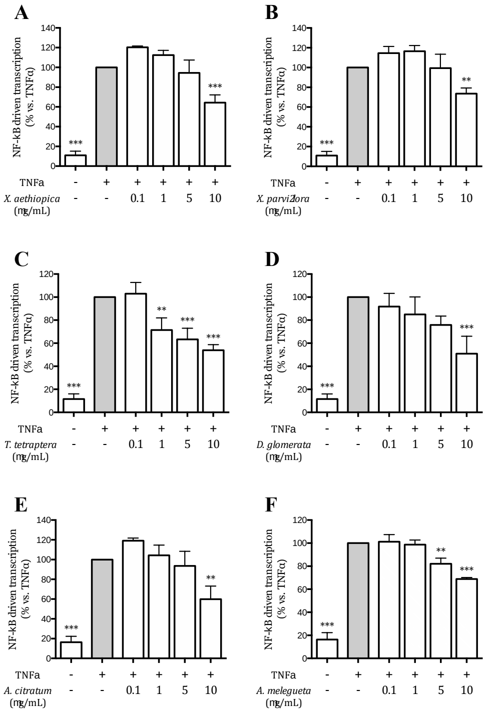

Figure 2. Effect of the extracts on the NF-κB driven transcription in GES-1 cells. Data are expressed as percentage versus the stimulated control, which is arbitrarily assigned the value 100%. ** $p < 0.01$; *** $p < 0.001$.

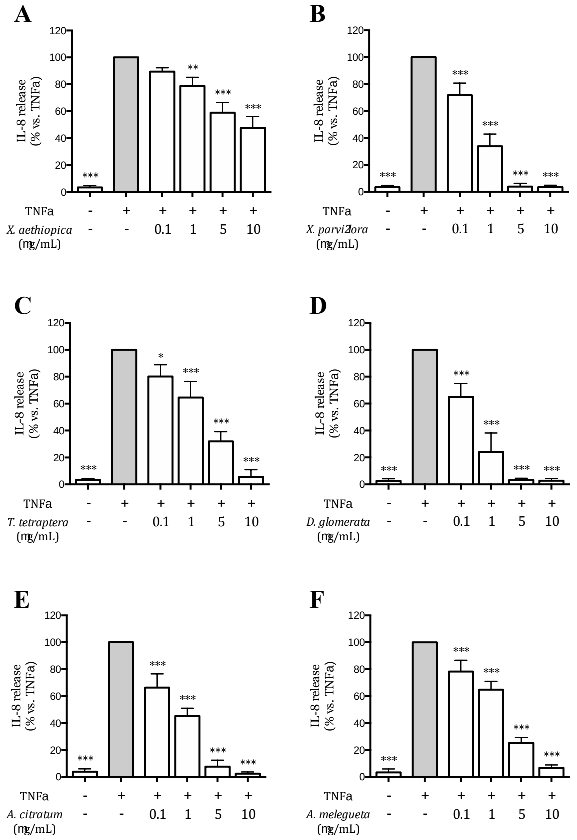

Figure 3. Effect of the extracts on the IL-8 release in AGS cells. Data are expressed as percentage versus the stimulated control, which is arbitrarily assigned the value 100%. * $p < 0.05$; ** $p < 0.01$; *** $p < 0.001$.

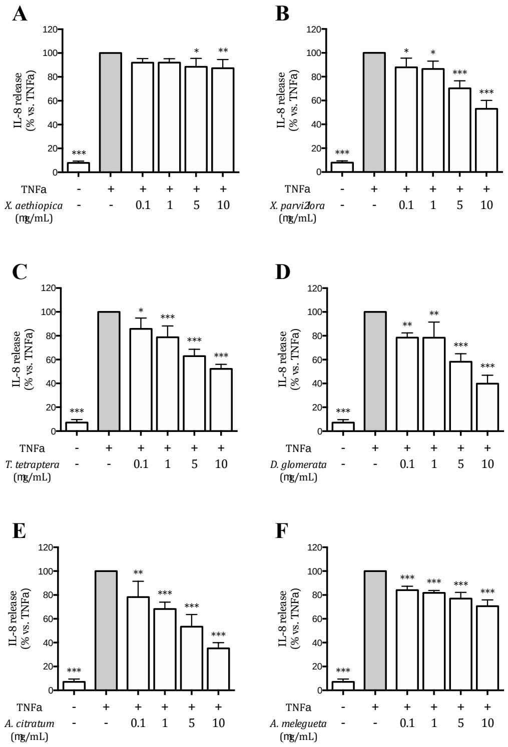

Figure S4. Effect of the extracts on the IL-8 release in GES-1 cells. Data are expressed as percentage versus the stimulated control, which is arbitrarily assigned the value 100%. * $p < 0.05$; ** $p < 0.01$; *** $p < 0.001$.

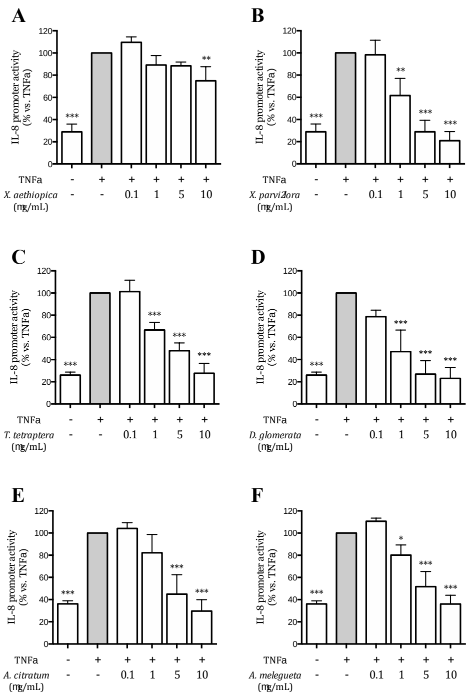

Figure S5. Effect of the extracts on the IL-8 promoter activity in AGS cells. Data are expressed as percentage versus the stimulated control, which is arbitrarily assigned the value 100%. * $p < 0.05$; ** $p < 0.01$; *** $p < 0.001$.

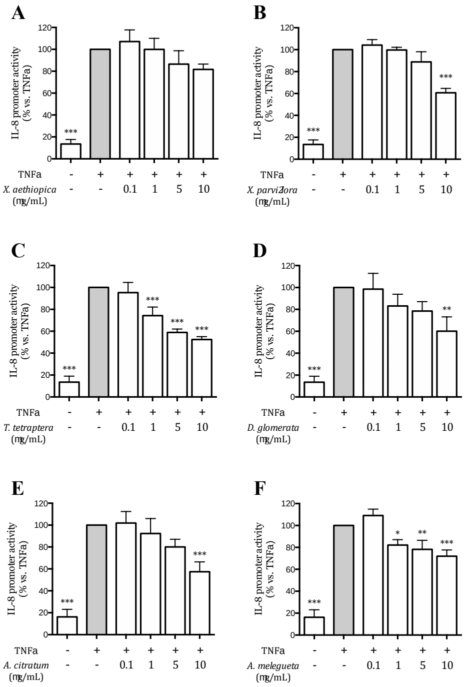

Figure S6. Effect of the extracts on the IL-8 promoter activity in GES-1 cells. Data are expressed as percentage versus the stimulated control, which is arbitrarily assigned the value 100%. * $p < 0.05$; ** $p < 0.01$; *** $p < 0.001$.

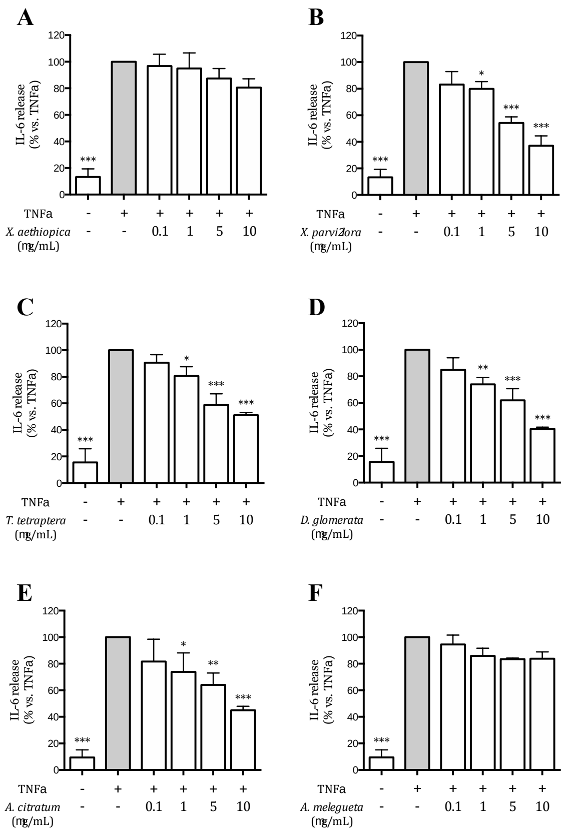

Figure S7. Effect of the extracts on the IL-6 release in GES-1 cells. Data are expressed as percentage versus the stimulated control, which is arbitrarily assigned the value 100%. * $p < 0.05$; ** $p < 0.01$; *** $p < 0.001$.
